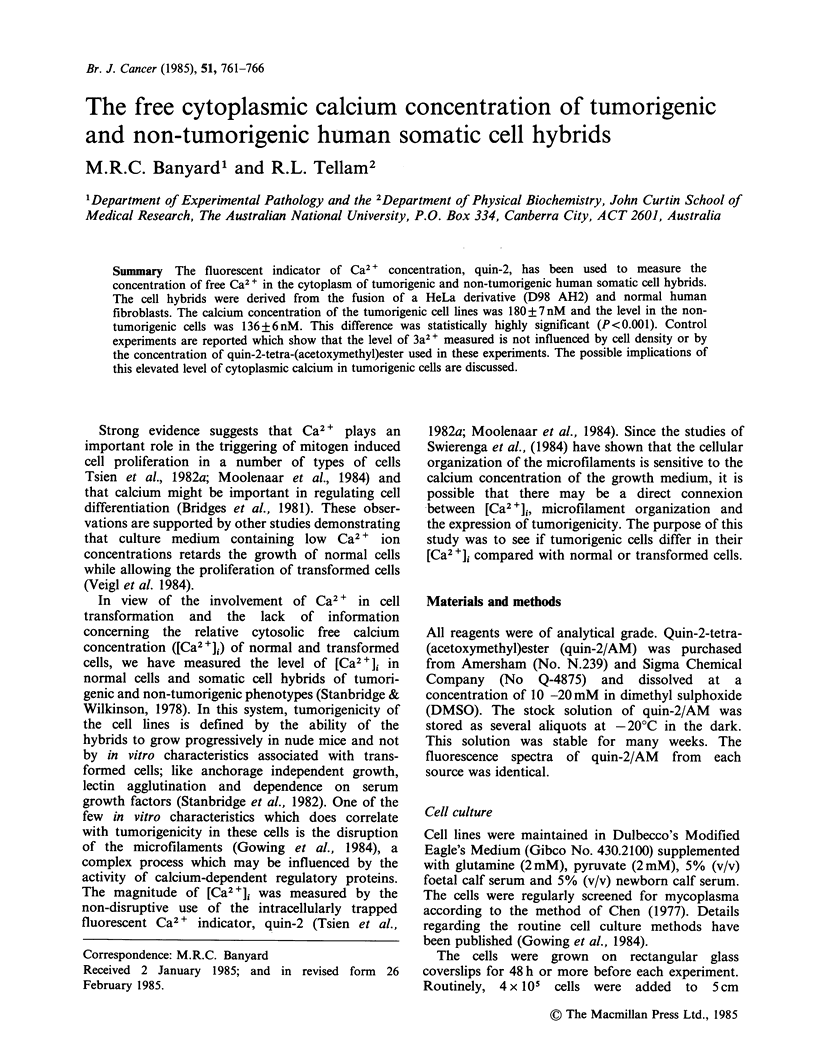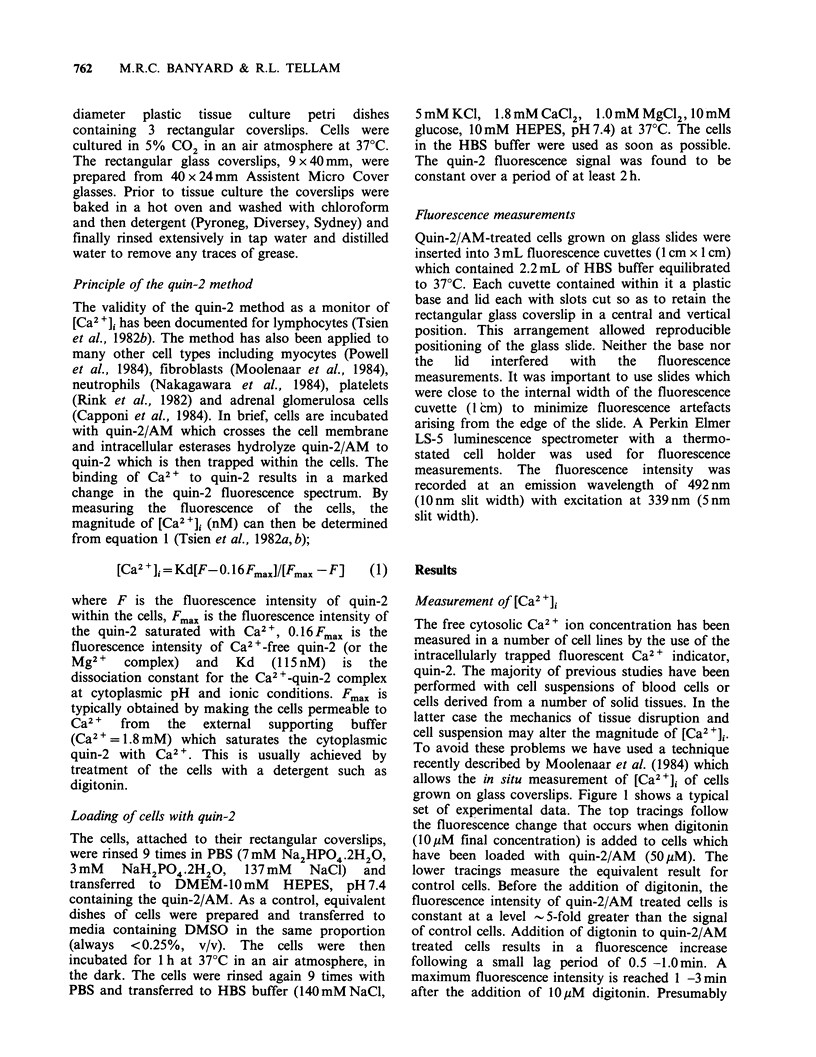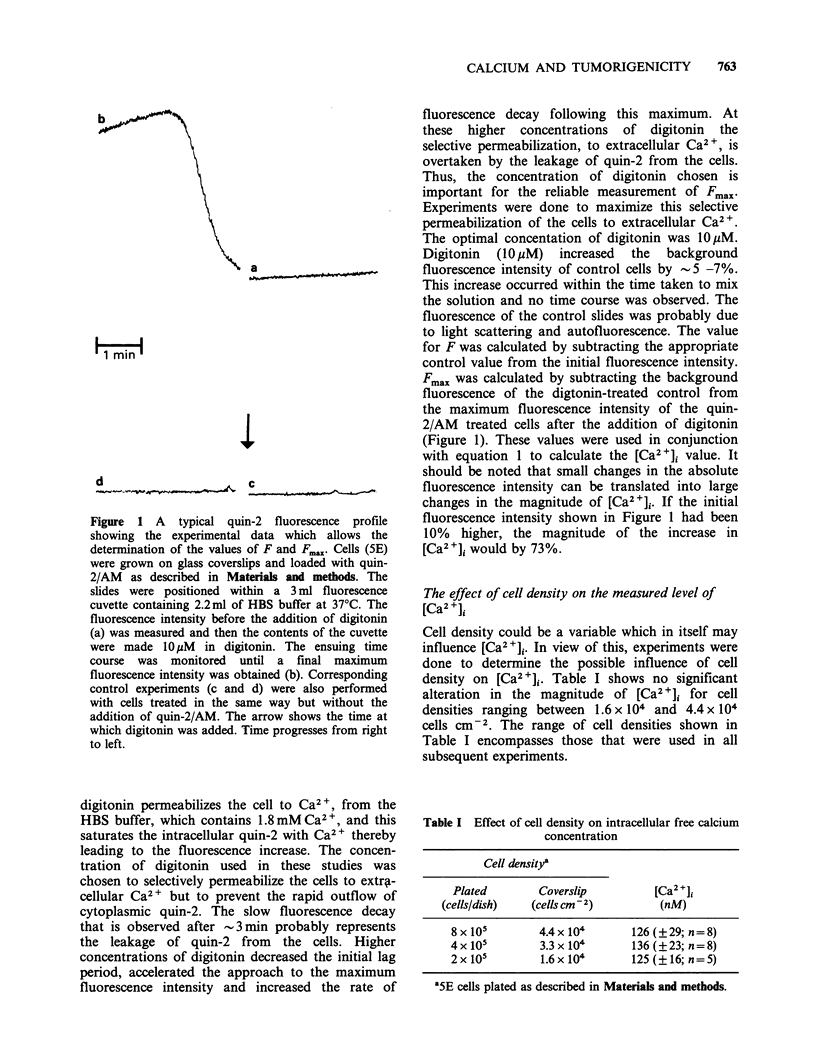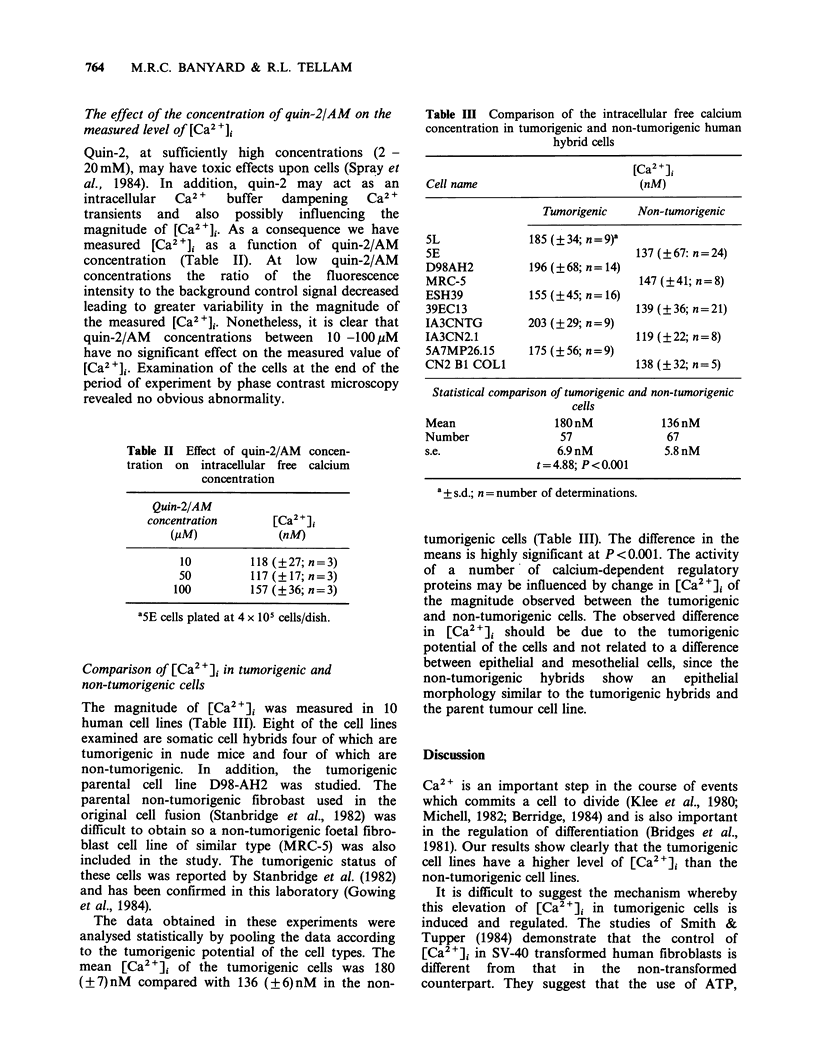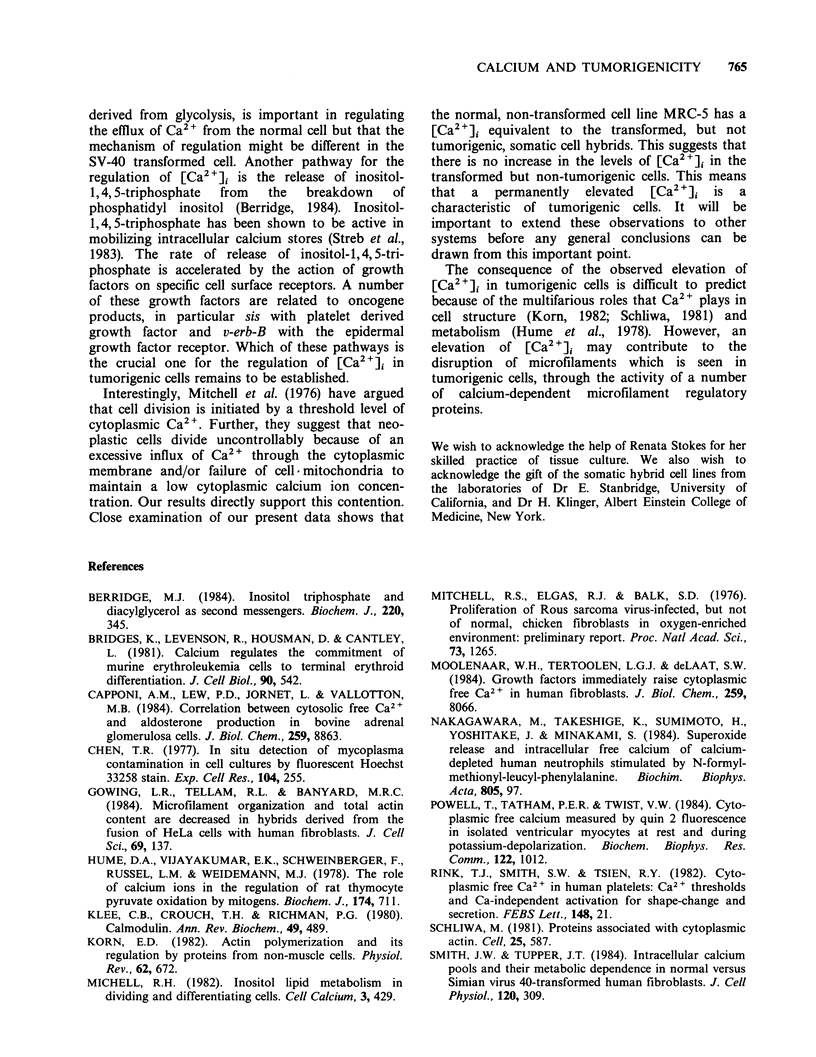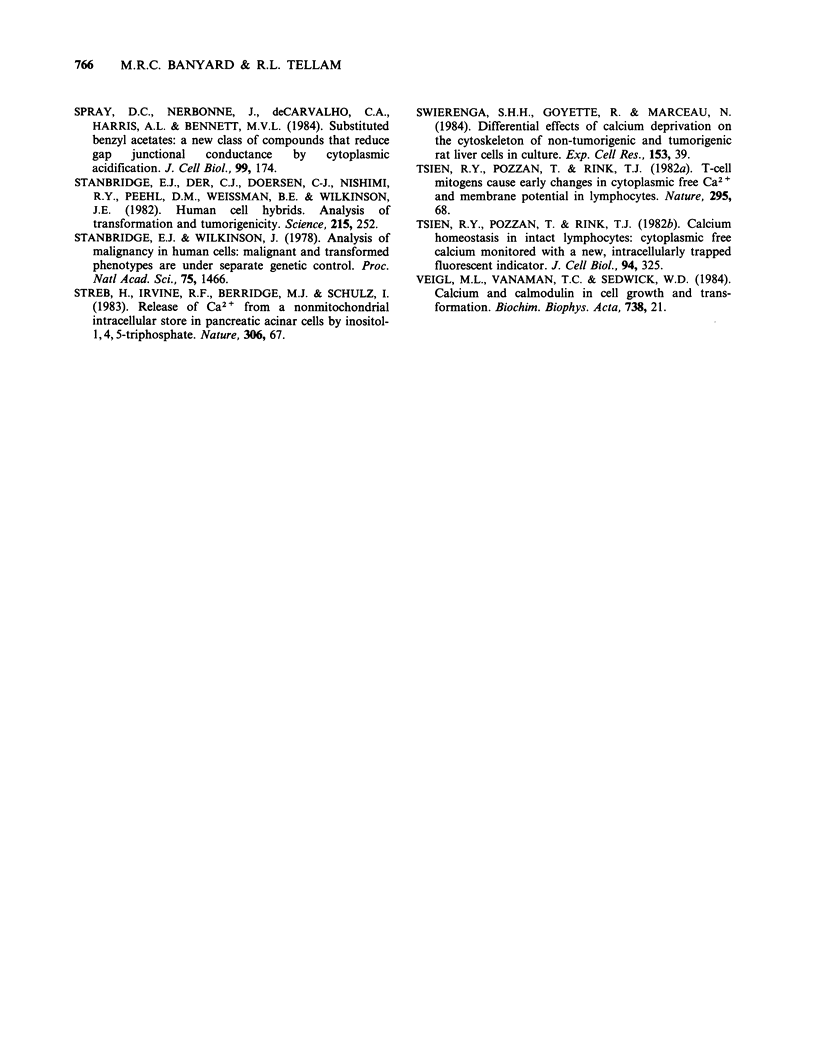# The free cytoplasmic calcium concentration of tumorigenic and non-tumorigenic human somatic cell hybrids.

**DOI:** 10.1038/bjc.1985.119

**Published:** 1985-06

**Authors:** M. R. Banyard, R. L. Tellam

## Abstract

The fluorescent indicator of Ca2+ concentration, quin-2, has been used to measure the concentration of free Ca2+ in the cytoplasm of tumorigenic and non-tumorigenic human somatic cell hybrids. The cell hybrids were derived from the fusion of a HeLa derivative (D98 AH2) and normal human fibroblasts. The calcium concentration of the tumorigenic cell lines was 180 +/- 7nM and the level in the non-tumorigenic cells was 136 +/- 6nM. This difference was statistically highly significant (P less than 0.001). Control experiments are reported which show that the level of 3a2+ measured is not influenced by cell density or by the concentration of quin-2-tetra-(acetoxymethyl)ester used in these experiments. The possible implications of this elevated level of cytoplasmic calcium in tumorigenic cells are discussed.


*Br. J. Cancer* (1985), **51**, 761–766

### The free cytoplasmic calcium concentration of tumorigenic and non-tumorigenic human somatic cell hybrids

M.R.C. Banyard[1] and R.L. Tellam[2]

[1]*Department of Experimental Pathology and the* [2]*Department of Physical Biochemistry, John Curtin School of Medical Research, The Australian National University, P.O. Box 334, Canberra City, ACT 2601, Australia*

**Summary** The fluorescent indicator of $Ca^{2+}$ concentration, quin-2, has been used to measure the concentration of free $Ca^{2+}$ in the cytoplasm of tumorigenic and non-tumorigenic human somatic cell hybrids. The cell hybrids were derived from the fusion of a HeLa derivative (D98 AH2) and normal human fibroblasts. The calcium concentration of the tumorigenic cell lines was $180 \pm 7$ nM and the level in the non-tumorigenic cells was $136 \pm 6$ nM. This difference was statistically highly significant ($P < 0.001$). Control experiments are reported which show that the level of $3a^{2+}$ measured is not influenced by cell density or by the concentration of quin-2-tetra-(acetoxymethyl)ester used in these experiments. The possible implications of this elevated level of cytoplasmic calcium in tumorigenic cells are discussed.

Strong evidence suggests that $Ca^{2+}$ plays an important role in the triggering of mitogen induced cell proliferation in a number of types of cells Tsien *et al.*, 1982*a*; Moolenaar *et al.*, 1984) and that calcium might be important in regulating cell differentiation (Bridges *et al.*, 1981). These observations are supported by other studies demonstrating that culture medium containing low $Ca^{2+}$ ion concentrations retards the growth of normal cells while allowing the proliferation of transformed cells (Veigl *et al.* 1984).

In view of the involvement of $Ca^{2+}$ in cell transformation and the lack of information concerning the relative cytosolic free calcium concentration ($[Ca^{2+}]_i$) of normal and transformed cells, we have measured the level of $[Ca^{2+}]_i$ in normal cells and somatic cell hybrids of tumorigenic and non-tumorigenic phenotypes (Stanbridge & Wilkinson, 1978). In this system, tumorigenicity of the cell lines is defined by the ability of the hybrids to grow progressively in nude mice and not by *in vitro* characteristics associated with transformed cells; like anchorage independent growth, lectin agglutination and dependence on serum growth factors (Stanbridge *et al.*, 1982). One of the few *in vitro* characteristics which does correlate with tumorigenicity in these cells is the disruption of the microfilaments (Gowing *et al.*, 1984), a complex process which may be influenced by the activity of calcium-dependent regulatory proteins. The magnitude of $[Ca^{2+}]_i$ was measured by the non-disruptive use of the intracellularly trapped fluorescent $Ca^{2+}$ indicator, quin-2 (Tsien *et al.*, 1982*a*; Moolenaar *et al.*, 1984). Since the studies of Swierenga *et al.*, (1984) have shown that the cellular organization of the microfilaments is sensitive to the calcium concentration of the growth medium, it is possible that there may be a direct connexion between $[Ca^{2+}]_i$, microfilament organization and the expression of tumorigenicity. The purpose of this study was to see if tumorigenic cells differ in their $[Ca^{2+}]_i$ compared with normal or transformed cells.

Correspondence: M.R.C. Banyard
Received 2 January 1985; and in revised form 26 February 1985.

### Materials and methods

All reagents were of analytical grade. Quin-2-tetra-(acetoxymethyl)ester (quin-2/AM) was purchased from Amersham (No. N.239) and Sigma Chemical Company (No Q-4875) and dissolved at a concentration of 10 –20 mM in dimethyl sulphoxide (DMSO). The stock solution of quin-2/AM was stored as several aliquots at −20°C in the dark. This solution was stable for many weeks. The fluorescence spectra of quin-2/AM from each source was identical.

### *Cell culture*

Cell lines were maintained in Dulbecco's Modified Eagle's Medium (Gibco No. 430.2100) supplemented with glutamine (2 mM), pyruvate (2 mM), 5% (v/v) foetal calf serum and 5% (v/v) newborn calf serum. The cells were regularly screened for mycoplasma according to the method of Chen (1977). Details regarding the routine cell culture methods have been published (Gowing *et al.*, 1984).

The cells were grown on rectangular glass coverslips for 48 h or more before each experiment. Routinely, $4 \times 10^5$ cells were added to 5 cm

© The Macmillan Press Ltd., 1985

diameter plastic tissue culture petri dishes containing 3 rectangular coverslips. Cells were cultured in 5% $CO_2$ in an air atmosphere at 37°C. The rectangular glass coverslips, 9 × 40 mm, were prepared from 40 × 24 mm Assistent Micro Cover glasses. Prior to tissue culture the coverslips were baked in a hot oven and washed with chloroform and then detergent (Pyroneg, Diversey, Sydney) and finally rinsed extensively in tap water and distilled water to remove any traces of grease.

*Principle of the quin-2 method*

The validity of the quin-2 method as a monitor of $[Ca^{2+}]_i$ has been documented for lymphocytes (Tsien *et al.*, 1982*b*). The method has also been applied to many other cell types including myocytes (Powell *et al.*, 1984), fibroblasts (Moolenaar *et al.*, 1984), neutrophils (Nakagawara *et al.*, 1984), platelets (Rink *et al.*, 1982) and adrenal glomerulosa cells (Capponi *et al.*, 1984). In brief, cells are incubated with quin-2/AM which crosses the cell membrane and intracellular esterases hydrolyze quin-2/AM to quin-2 which is then trapped within the cells. The binding of $Ca^{2+}$ to quin-2 results in a marked change in the quin-2 fluorescence spectrum. By measuring the fluorescence of the cells, the magnitude of $[Ca^{2+}]_i$ (nM) can then be determined from equation 1 (Tsien *et al.*, 1982*a*, *b*);

$$[Ca^{2+}]_i = \mathrm{Kd}[F - 0.16F_{max}]/[F_{max} - F] \quad (1)$$

where $F$ is the fluorescence intensity of quin-2 within the cells, $F_{max}$ is the fluorescence intensity of the quin-2 saturated with $Ca^{2+}$, $0.16F_{max}$ is the fluorescence intensity of $Ca^{2+}$-free quin-2 (or the $Mg^{2+}$ complex) and Kd (115 nM) is the dissociation constant for the $Ca^{2+}$-quin-2 complex at cytoplasmic pH and ionic conditions. $F_{max}$ is typically obtained by making the cells permeable to $Ca^{2+}$ from the external supporting buffer ($Ca^{2+} = 1.8$ mM) which saturates the cytoplasmic quin-2 with $Ca^{2+}$. This is usually achieved by treatment of the cells with a detergent such as digitonin.

*Loading of cells with quin-2*

The cells, attached to their rectangular coverslips, were rinsed 9 times in PBS (7 mM $Na_2HPO_4.2H_2O$, 3 mM $NaH_2PO_4.2H_2O$, 137 mM NaCl) and transferred to DMEM-10 mM HEPES, pH 7.4 containing the quin-2/AM. As a control, equivalent dishes of cells were prepared and transferred to media containing DMSO in the same proportion (always <0.25%, v/v). The cells were then incubated for 1 h at 37°C in an air atmosphere, in the dark. The cells were rinsed again 9 times with PBS and transferred to HBS buffer (140 mM NaCl, 5 mM KCl, 1.8 mM $CaCl_2$, 1.0 mM $MgCl_2$, 10 mM glucose, 10 mM HEPES, pH 7.4) at 37°C. The cells in the HBS buffer were used as soon as possible. The quin-2 fluorescence signal was found to be constant over a period of at least 2 h.

*Fluorescence measurements*

Quin-2/AM-treated cells grown on glass slides were inserted into 3 mL fluorescence cuvettes (1 cm × 1 cm) which contained 2.2 mL of HBS buffer equilibrated to 37°C. Each cuvette contained within it a plastic base and lid each with slots cut so as to retain the rectangular glass coverslip in a central and vertical position. This arrangement allowed reproducible positioning of the glass slide. Neither the base nor the lid interfered with the fluorescence measurements. It was important to use slides which were close to the internal width of the fluorescence cuvette (1 cm) to minimize fluorescence artefacts arising from the edge of the slide. A Perkin Elmer LS-5 luminescence spectrometer with a thermostated cell holder was used for fluorescence measurements. The fluorescence intensity was recorded at an emission wavelength of 492 nm (10 nm slit width) with excitation at 339 nm (5 nm slit width).

### Results

*Measurement of* $[Ca^{2+}]_i$

The free cytosolic $Ca^{2+}$ ion concentration has been measured in a number of cell lines by the use of the intracellularly trapped fluorescent $Ca^{2+}$ indicator, quin-2. The majority of previous studies have been performed with cell suspensions of blood cells or cells derived from a number of solid tissues. In the latter case the mechanics of tissue disruption and cell suspension may alter the magnitude of $[Ca^{2+}]_i$. To avoid these problems we have used a technique recently described by Moolenaar *et al.* (1984) which allows the *in situ* measurement of $[Ca^{2+}]_i$ of cells grown on glass coverslips. Figure 1 shows a typical set of experimental data. The top tracings follow the fluorescence change that occurs when digitonin (10 $\mu$M final concentration) is added to cells which have been loaded with quin-2/AM (50 $\mu$M). The lower tracings measure the equivalent result for control cells. Before the addition of digitonin, the fluorescence intensity of quin-2/AM treated cells is constant at a level ~5-fold greater than the signal of control cells. Addition of digtonin to quin-2/AM treated cells results in a fluorescence increase following a small lag period of 0.5 -1.0 min. A maximum fluorescence intensity is reached 1 -3 min after the addition of 10 $\mu$M digitonin. Presumably

**Figure 1** A typical quin-2 fluorescence profile showing the experimental data which allows the determination of the values of $F$ and $F_{max}$. Cells (5E) were grown on glass coverslips and loaded with quin-2/AM as described in **Materials and methods**. The slides were positioned within a 3 ml fluorescence cuvette containing 2.2 ml of HBS buffer at 37°C. The fluorescence intensity before the addition of digitonin (a) was measured and then the contents of the cuvette were made 10 $\mu$M in digitonin. The ensuing time course was monitored until a final maximum fluorescence intensity was obtained (b). Corresponding control experiments (c and d) were also performed with cells treated in the same way but without the addition of quin-2/AM. The arrow shows the time at which digitonin was added. Time progresses from right to left.

digitonin permeabilizes the cell to $Ca^{2+}$, from the HBS buffer, which contains 1.8 mM $Ca^{2+}$, and this saturates the intracellular quin-2 with $Ca^{2+}$ thereby leading to the fluorescence increase. The concentration of digitonin used in these studies was chosen to selectively permeabilize the cells to extracellular $Ca^{2+}$ but to prevent the rapid outflow of cytoplasmic quin-2. The slow fluorescence decay that is observed after ~3 min probably represents the leakage of quin-2 from the cells. Higher concentrations of digitonin decreased the initial lag period, accelerated the approach to the maximum fluorescence intensity and increased the rate of fluorescence decay following this maximum. At these higher concentrations of digitonin the selective permeabilization, to extracellular $Ca^{2+}$, is overtaken by the leakage of quin-2 from the cells. Thus, the concentration of digitonin chosen is important for the reliable measurement of $F_{max}$. Experiments were done to maximize this selective permeabilization of the cells to extracellular $Ca^{2+}$. The optimal concentation of digitonin was 10 $\mu$M. Digitonin (10 $\mu$M) increased the background fluorescence intensity of control cells by ~5 –7%. This increase occurred within the time taken to mix the solution and no time course was observed. The fluorescence of the control slides was probably due to light scattering and autofluorescence. The value for $F$ was calculated by subtracting the appropriate control value from the initial fluorescence intensity. $F_{max}$ was calculated by subtracting the background fluorescence of the digtonin-treated control from the maximum fluorescence intensity of the quin-2/AM treated cells after the addition of digitonin (Figure 1). These values were used in conjunction with equation 1 to calculate the $[Ca^{2+}]_i$ value. It should be noted that small changes in the absolute fluorescence intensity can be translated into large changes in the magnitude of $[Ca^{2+}]_i$. If the initial fluorescence intensity shown in Figure 1 had been 10% higher, the magnitude of the increase in $[Ca^{2+}]_i$ would by 73%.

### *The effect of cell density on the measured level of* $[Ca^{2+}]_i$

Cell density could be a variable which in itself may influence $[Ca^{2+}]_i$. In view of this, experiments were done to determine the possible influence of cell density on $[Ca^{2+}]_i$. Table I shows no significant alteration in the magnitude of $[Ca^{2+}]_i$ for cell densities ranging between $1.6 \times 10^4$ and $4.4 \times 10^4$ cells $cm^{-2}$. The range of cell densities shown in Table I encompasses those that were used in all subsequent experiments.

**Table I** Effect of cell density on intracellular free calcium concentration

| *Cell density*[a] | | |
|---|---|---|
| *Plated (cells/dish)* | *Coverslip (cells cm$^{-2}$)* | $[Ca^{2+}]_i$ (*nM*) |
| $8 \times 10^5$ | $4.4 \times 10^4$ | 126 ($\pm$29; $n$=8) |
| $4 \times 10^5$ | $3.3 \times 10^4$ | 136 ($\pm$23; $n$=8) |
| $2 \times 10^5$ | $1.6 \times 10^4$ | 125 ($\pm$16; $n$=5) |

[a]5E cells plated as described in **Materials and methods**.

*The effect of the concentration of quin-2/AM on the measured level of* $[Ca^{2+}]_i$

Quin-2, at sufficiently high concentrations (2 – 20 mM), may have toxic effects upon cells (Spray *et al.*, 1984). In addition, quin-2 may act as an intracellular $Ca^{2+}$ buffer dampening $Ca^{2+}$ transients and also possibly influencing the magnitude of $[Ca^{2+}]_i$. As a consequence we have measured $[Ca^{2+}]_i$ as a function of quin-2/AM concentration (Table II). At low quin-2/AM concentrations the ratio of the fluorescence intensity to the background control signal decreased leading to greater variability in the magnitude of the measured $[Ca^{2+}]_i$. Nonetheless, it is clear that quin-2/AM concentrations between 10 –100 μM have no significant effect on the measured value of $[Ca^{2+}]_i$. Examination of the cells at the end of the period of experiment by phase contrast microscopy revealed no obvious abnormality.

**Table II** Effect of quin-2/AM concentration on intracellular free calcium concentration

| *Quin-2/AM concentration (μM)* | $[Ca^{2+}]_i$ *(nM)* |
|---|---|
| 10 | 118 (±27; *n*=3) |
| 50 | 117 (±17; *n*=3) |
| 100 | 157 (±36; *n*=3) |

[a]5E cells plated at 4 × $10^5$ cells/dish.

*Comparison of* $[Ca^{2+}]_i$ *in tumorigenic and non-tumorigenic cells*

The magnitude of $[Ca^{2+}]_i$ was measured in 10 human cell lines (Table III). Eight of the cell lines examined are somatic cell hybrids four of which are tumorigenic in nude mice and four of which are non-tumorigenic. In addition, the tumorigenic parental cell line D98-AH2 was studied. The parental non-tumorigenic fibrobast used in the original cell fusion (Stanbridge *et al.*, 1982) was difficult to obtain so a non-tumorigenic foetal fibroblast cell line of similar type (MRC-5) was also included in the study. The tumorigenic status of these cells was reported by Stanbridge *et al.* (1982) and has been confirmed in this laboratory (Gowing *et al.*, 1984).

The data obtained in these experiments were analysed statistically by pooling the data according to the tumorigenic potential of the cell types. The mean $[Ca^{2+}]_i$ of the tumorigenic cells was 180 (±7) nM compared with 136 (±6) nM in the non-tumorigenic cells (Table III). The difference in the means is highly significant at $P<0.001$. The activity of a number of calcium-dependent regulatory proteins may be influenced by change in $[Ca^{2+}]_i$ of the magnitude observed between the tumorigenic and non-tumorigenic cells. The observed difference in $[Ca^{2+}]_i$ should be due to the tumorigenic potential of the cells and not related to a difference between epithelial and mesothelial cells, since the non-tumorigenic hybrids show an epithelial morphology similar to the tumorigenic hybrids and the parent tumour cell line.

**Table III** Comparison of the intracellular free calcium concentration in tumorigenic and non-tumorigenic human hybrid cells

| *Cell name* | $[Ca^{2+}]_i$ *(nM)* *Tumorigenic* | *Non-tumorigenic* |
|---|---|---|
| 5L | 185 (±34; *n*=9)[a] | |
| 5E | | 137 (±67: *n*=24) |
| D98AH2 | 196 (±68; *n*=14) | |
| MRC-5 | | 147 (±41; *n*=8) |
| ESH39 | 155 (±45; *n*=16) | |
| 39EC13 | | 139 (±36; *n*=21) |
| IA3CNTG | 203 (±29; *n*=9) | |
| IA3CN2.1 | | 119 (±22; *n*=8) |
| 5A7MP26.15 | 175 (±56; *n*=9) | |
| CN2 B1 COL1 | | 138 (±32; *n*=5) |
| *Statistical comparison of tumorigenic and non-tumorigenic cells* | | |
| Mean | 180 nM | 136 nM |
| Number | 57 | 67 |
| s.e. | 6.9 nM | 5.8 nM |
| | $t=4.88$; $P<0.001$ | |

[a]±s.d.; *n*=number of determinations.

### Discussion

$Ca^{2+}$ is an important step in the course of events which commits a cell to divide (Klee *et al.*, 1980; Michell, 1982; Berridge, 1984) and is also important in the regulation of differentiation (Bridges *et al.*, 1981). Our results show clearly that the tumorigenic cell lines have a higher level of $[Ca^{2+}]_i$ than the non-tumorigenic cell lines.

It is difficult to suggest the mechanism whereby this elevation of $[Ca^{2+}]_i$ in tumorigenic cells is induced and regulated. The studies of Smith & Tupper (1984) demonstrate that the control of $[Ca^{2+}]_i$ in SV-40 transformed human fibroblasts is different from that in the non-transformed counterpart. They suggest that the use of ATP, derived from glycolysis, is important in regulating the efflux of $Ca^{2+}$ from the normal cell but that the mechanism of regulation might be different in the SV-40 transformed cell. Another pathway for the regulation of $[Ca^{2+}]_i$ is the release of inositol-1,4,5-triphosphate from the breakdown of phosphatidyl inositol (Berridge, 1984). Inositol-1,4,5-triphosphate has been shown to be active in mobilizing intracellular calcium stores (Streb *et al.*, 1983). The rate of release of inositol-1,4,5-triphosphate is accelerated by the action of growth factors on specific cell surface receptors. A number of these growth factors are related to oncogene products, in particular *sis* with platelet derived growth factor and *v-erb-B* with the epidermal growth factor receptor. Which of these pathways is the crucial one for the regulation of $[Ca^{2+}]_i$ in tumorigenic cells remains to be established.

Interestingly, Mitchell *et al.* (1976) have argued that cell division is initiated by a threshold level of cytoplasmic $Ca^{2+}$. Further, they suggest that neoplastic cells divide uncontrollably because of an excessive influx of $Ca^{2+}$ through the cytoplasmic membrane and/or failure of cell·mitochondria to maintain a low cytoplasmic calcium ion concentration. Our results directly support this contention. Close examination of our present data shows that the normal, non-transformed cell line MRC-5 has a $[Ca^{2+}]_i$ equivalent to the transformed, but not tumorigenic, somatic cell hybrids. This suggests that there is no increase in the levels of $[Ca^{2+}]_i$ in the transformed but non-tumorigenic cells. This means that a permanently elevated $[Ca^{2+}]_i$ is a characteristic of tumorigenic cells. It will be important to extend these observations to other systems before any general conclusions can be drawn from this important point.

The consequence of the observed elevation of $[Ca^{2+}]_i$ in tumorigenic cells is difficult to predict because of the multifarious roles that $Ca^{2+}$ plays in cell structure (Korn, 1982; Schliwa, 1981) and metabolism (Hume *et al.*, 1978). However, an elevation of $[Ca^{2+}]_i$ may contribute to the disruption of microfilaments which is seen in tumorigenic cells, through the activity of a number of calcium-dependent microfilament regulatory proteins.

We wish to acknowledge the help of Renata Stokes for her skilled practice of tissue culture. We also wish to acknowledge the gift of the somatic hybrid cell lines from the laboratories of Dr E. Stanbridge, University of California, and Dr H. Klinger, Albert Einstein College of Medicine, New York.

### References

BERRIDGE, M.J. (1984). Inositol triphosphate and diacylglycerol as second messengers. *Biochem. J.*, **220**, 345.

BRIDGES, K., LEVENSON, R., HOUSMAN, D. & CANTLEY, L. (1981). Calcium regulates the commitment of murine erythroleukemia cells to terminal erythroid differentiation. *J. Cell Biol.*, **90**, 542.

CAPPONI, A.M., LEW, P.D., JORNET, L. & VALLOTTON, M.B. (1984). Correlation between cytosolic free $Ca^{2+}$ and aldosterone production in bovine adrenal glomerulosa cells. *J. Biol. Chem.*, **259**, 8863.

CHEN, T.R. (1977). In situ detection of mycoplasma contamination in cell cultures by fluorescent Hoechst 33258 stain. *Exp. Cell Res.*, **104**, 255.

GOWING, L.R., TELLAM, R.L. & BANYARD, M.R.C. (1984). Microfilament organization and total actin content are decreased in hybrids derived from the fusion of HeLa cells with human fibroblasts. *J. Cell Sci.*, **69**, 137.

HUME, D.A., VIJAYAKUMAR, E.K., SCHWEINBERGER, F., RUSSEL, L.M. & WEIDEMANN, M.J. (1978). The role of calcium ions in the regulation of rat thymocyte pyruvate oxidation by mitogens. *Biochem. J.*, **174**, 711.

KLEE, C.B., CROUCH, T.H. & RICHMAN, P.G. (1980). Calmodulin. *Ann. Rev. Biochem.*, **49**, 489.

KORN, E.D. (1982). Actin polymerization and its regulation by proteins from non-muscle cells. *Physiol. Rev.*, **62**, 672.

MICHELL, R.H. (1982). Inositol lipid metabolism in dividing and differentiating cells. *Cell Calcium*, **3**, 429.

MITCHELL, R.S., ELGAS, R.J. & BALK, S.D. (1976). Proliferation of Rous sarcoma virus-infected, but not of normal, chicken fibroblasts in oxygen-enriched environment: preliminary report. *Proc. Natl Acad. Sci.*, **73**, 1265.

MOOLENAAR, W.H., TERTOOLEN, L.G.J. & deLAAT, S.W. (1984). Growth factors immediately raise cytoplasmic free $Ca^{2+}$ in human fibroblasts. *J. Biol. Chem.*, **259**, 8066.

NAKAGAWARA, M., TAKESHIGE, K., SUMIMOTO, H., YOSHITAKE, J. & MINAKAMI, S. (1984). Superoxide release and intracellular free calcium of calcium-depleted human neutrophils stimulated by N-formyl-methionyl-leucyl-phenylalanine. *Biochim. Biophys. Acta*, **805**, 97.

POWELL, T., TATHAM, P.E.R. & TWIST, V.W. (1984). Cytoplasmic free calcium measured by quin 2 fluorescence in isolated ventricular myocytes at rest and during potassium-depolarization. *Biochem. Biophys. Res. Comm.*, **122**, 1012.

RINK, T.J., SMITH, S.W. & TSIEN, R.Y. (1982). Cytoplasmic free $Ca^{2+}$ in human platelets: $Ca^{2+}$ thresholds and Ca-independent activation for shape-change and secretion. *FEBS Lett.*, **148**, 21.

SCHLIWA, M. (1981). Proteins associated with cytoplasmic actin. *Cell*, **25**, 587.

SMITH, J.W. & TUPPER, J.T. (1984). Intracellular calcium pools and their metabolic dependence in normal versus Simian virus 40-transformed human fibroblasts. *J. Cell Physiol.*, **120**, 309.

SPRAY, D.C., NERBONNE, J., deCARVALHO, C.A., HARRIS, A.L. & BENNETT, M.V.L. (1984). Substituted benzyl acetates: a new class of compounds that reduce gap junctional conductance by cytoplasmic acidification. *J. Cell Biol.*, **99**, 174.

STANBRIDGE, E.J., DER, C.J., DOERSEN, C-J., NISHIMI, R.Y., PEEHL, D.M., WEISSMAN, B.E. & WILKINSON, J.E. (1982). Human cell hybrids. Analysis of transformation and tumorigenicity. *Science*, **215**, 252.

STANBRIDGE, E.J. & WILKINSON, J. (1978). Analysis of malignancy in human cells: malignant and transformed phenotypes are under separate genetic control. *Proc. Natl Acad. Sci.*, **75**, 1466.

STREB, H., IRVINE, R.F., BERRIDGE, M.J. & SCHULZ, I. (1983). Release of $Ca^{2+}$ from a nonmitochondrial intracellular store in pancreatic acinar cells by inositol-1,4,5-triphosphate. *Nature*, **306**, 67.

SWIERENGA, S.H.H., GOYETTE, R. & MARCEAU, N. (1984). Differential effects of calcium deprivation on the cytoskeleton of non-tumorigenic and tumorigenic rat liver cells in culture. *Exp. Cell Res.*, **153**, 39.

TSIEN, R.Y., POZZAN, T. & RINK, T.J. (1982*a*). T-cell mitogens cause early changes in cytoplasmic free $Ca^{2+}$ and membrane potential in lymphocytes. *Nature*, **295**, 68.

TSIEN, R.Y., POZZAN, T. & RINK, T.J. (1982*b*). Calcium homeostasis in intact lymphocytes: cytoplasmic free calcium monitored with a new, intracellularly trapped fluorescent indicator. *J. Cell Biol.*, **94**, 325.

VEIGL, M.L., VANAMAN, T.C. & SEDWICK, W.D. (1984). Calcium and calmodulin in cell growth and transformation. *Biochim. Biophys. Acta*, **738**, 21.